# Systematic Review and Guidelines for Management of Scrotal Inguinal Hernias

**DOI:** 10.3389/jaws.2023.11195

**Published:** 2023-03-27

**Authors:** Hanh Minh Tran, Ian MacQueen, David Chen, Maarten Simons

**Affiliations:** ^1^ The Sydney Hernia Specialists Clinic, Sydney, NSW, Australia; ^2^ Lichtenstein Amid Hernia Clinic, University College Los Angeles, Los Angeles, CA, United States; ^3^ Onze Lieve Vrouwe Gasthuis (OLVG), Amsterdam, Netherlands

**Keywords:** systematic review, guidelines, inguinal hernia, management, scrotal

## Abstract

**Introduction:** Of the more than 20 million patients undergoing groin hernia repair annually worldwide, 6% are scrotal hernias in high resource countries rising to 67% in low resource countries which represents a heavy disease burden on relatively young men during their most productive period of life. There are many open questions concerning management of scrotal hernia. These guidelines aim to improve the care for scrotal hernia patients by reducing recurrence rates, chronic pain and infection.

**Methods:** After developing 19 key questions a systematic literature review was performed till 31 March 2021 for all relevant publications with search terms related to Scrotal Hernia. The articles were scored by all co-authors according to Oxford, SIGN and Grade methodologies. Statements and recommendations were formulated. Online Consensus meetings with 25 HerniaSurge members were organised with voting and grading Recommendations as “strong” (recommendations) or “weak” (suggestions) and by consensus, in some cases upgraded.

**Results:** Only 23 articles (two level 2 registry and 21 level 3–5) were selected. It is proposed to define scrotal hernia as an inguinal hernia which has descended into and causes any scrotal distortion. A new classification for scrotal hernias was proposed based on hernia size, SI for upper third thigh, SII for middle thigh and SIII for lower third thigh or below. Irreducibility is denoted with IR. Despite weak evidence antibiotic prophylaxis is recommended. Urinary catheterization is recommended (upgraded) in complex cases (S2-3) due to prolonged operative time. Scrotal hernia repairs have higher associated morbidity and mortality compared to non-complex groin hernia repairs irrespective of surgical experience. Open anterior (mesh) approach is commonest technique and suture techniques in low resource countries. For minimally invasive approaches, TAPP resulted in less conversion to open approach compared to TEP.

**Conclusion:** Although the evidence is scarce and often low quality scrotal hernia management guidelines aim to lead to better surgical outcomes irrespective of where patients live. This necessarily means a more tailored approach based on available resources and appropriate skills. The guidelines provide an impetus for future research where adoption of proposed classification will enable more meaningful comparison of different techniques for different hernia sizes.

## Introduction

Groin hernias represent a significant disease burden in the world with over 20 million hernia repairs being performed annually (1). Scrotal hernias represent a subset occurring in up to 6% in high resource countries but can occur in up to 67% of all presented cases in low resource countries (2). The latter group is of particular importance because they tend to occur in younger patients who are often the family bread winners with consequent negative personal, socioeconomic, and societal consequences. Factors contributing to the occurrence of scrotal hernias include delay in seeking healthcare, lack of access to healthcare, lack of financial means, medical comorbidities, illiteracy and lack of education, and fear of surgery (3).

Irrespective of available access to surgical intervention, scrotal hernias present significant challenges even to experienced surgeons because they have higher associated morbidity and mortality compared to non-complex groin hernia repairs. In this guidelines update, a systematic review was performed on available literature concerning management aspects of scrotal hernias. The HerniaSurge group developed these recommendations which are primarily consensus-based due to the low level of scientific evidence available for analysis.

## Methodology

Key Questions were formulated concerning pertinent aspects of the management of scrotal inguinal hernia.

**FIGURE 1:**
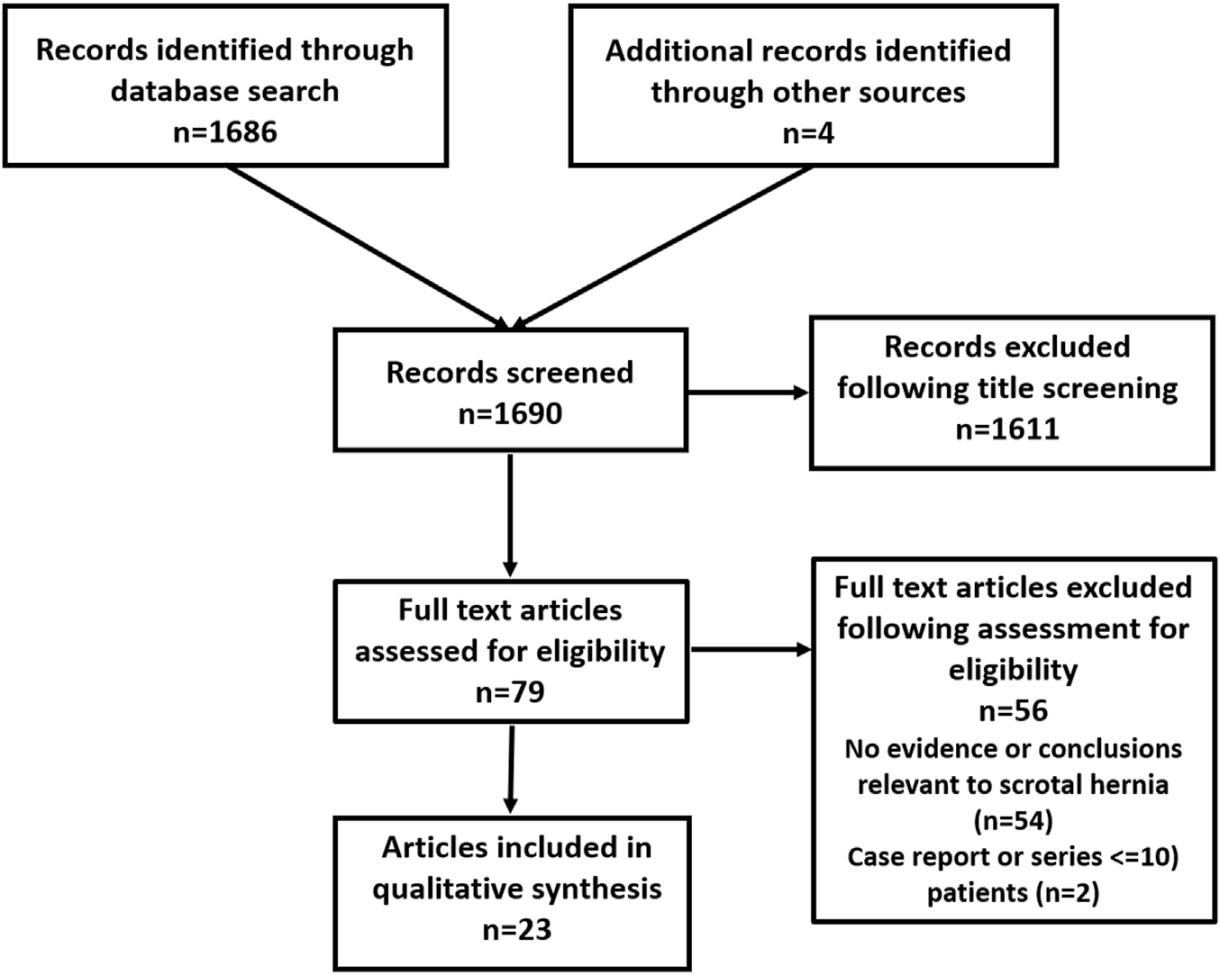
Flow chart of study inclusion for scrotal hernia.

Search terms used were “scrotal hernia” and “inguino-scrotal hernia” using Pubmed, Cochrane, Embase, Google Scholar up to 19 March 2021. Additionally, all bibliographies from included articles were cross-referenced. There were 1690 articles, and all of these were screened for relevance to answer the formulated key questions. Only articles with 10 or more cases were included in this review although occasional case reports detailing surgical techniques were included. A total of 23 articles were included according to SIGN criteria (1) with acceptable or high quality of assessment (Figure 1). Full articles were studied and rated by all team members: two Level 2 (4, 5), three Level 3 (6-8), fourteen Level 4 and four existing guidelines Level 5. The latter group include European Hernia Society (EHS) Guidelines in 2009 (9), International Endohernia Society (IEHS) Guidelines in 2011 (10), EHS Guidelines Update in 2014 (11), and Brazilian Hernia Society Guidelines in 2019 (12). Statements and recommendations were made depending on the strength of the evidence and some have been upgraded by the HerniaSurge Group by consensus.

### Definition of Scrotal Hernia

#### KQ1: Is there a Definition and System of Classification for Scrotal Hernia?

Recommendation: Scrotal hernia is defined as an inguinal hernia which has descended into and causes any distortion of the scrotum. It is suggested to denote Scrotal Hernia with an S and this can be subdivided into S1 (upper third thigh), S2 (middle third thigh) and S3 (lower third thigh/patellar). Measurements should be taken from the mid inguinal point to the lowest part of the scrotum in upright position. S(IR) is used to denote irreducible scrotal hernia.

Level of Evidence: Very Low.

Strength of recommendation: Weak.

Discussion: Unlike inguinal hernia where the EHS Classification is widely accepted, there is currently no generally accepted classification for scrotal hernias. The acceptance of the EHS Classification has largely resulted from its simplicity (i.e., L for lateral, M for medial hernias and the 1-3 for size). In this classification system, scrotal hernias are mostly classified as L3 and/or M3. The use of extent of scrotal descent has not been generally accepted and terms such as massive and giant inguino-scrotal hernia have been used without uniformity. Additionally, the complexity and associated operative and perioperative risks are not adequately presented in the current inguinal classification system (13). Therefore, a simplified, pragmatic, and clinically based classification system for scrotal hernia will potentially complete the EHS nomenclature for diagnosis, perioperative management and research. Sanders et al. (7) suggested a system of denoting the extent of scrotal descent by 10, 20 and 30 cm increments. This has the inherent limitation of assuming all adults having a fixed height which is not as applicable to people with different heights nor as representative of the worldwide variation amongst population groups. Trakarnsagna et al. (14) defined giant scrotal hernia as the hernia sac extending below the mid-inner thigh and suggested subdivision into Type I (under mid-inner thigh), Type II (from mid between mid-inner thigh and suprapatellar line) and Type III (under superior border of patellar bone). Ertem et al. (15) used a volumetric system of grading scrotal sac using CT scan during valsalva to assess size (0–500, 500–999,1000–1999, 2000–2999 and >3,000 mL). Recommendations for the operative technique based on hernia volume were made. This classification is impractical in most low resource countries which do not have consistent access to advanced imaging modalities and where scrotal hernias are most prevalent.

To supplement the current EHS inguinal hernia classification (13) a pragmatic classification for scrotal hernia is proposed to be added:

S1 = upper third thigh
S2 = middle third thigh
S3 = lower third thigh or below

It is important to denote whether the hernia is irreducible S(IR) as this will have implications on type of surgical repair to recommend.

### Risks of Complications

#### KQ2: Is there a Greater Risk of Complications Associated With Scrotal Hernia Repair?

Statement: Repair of scrotal hernia using whatever method has a higher risk of complications than non-scrotal inguinal hernia.

Level of Evidence: Low.

Discussion: Köckerling et al. (Kockerling et al., Hernia, 2020, 03: 1169–1181) reported results of 98,321 patients using data from HerniaMed Registry which includes data from 712 institutions up to 1 Feb 2019. There were 2710 scrotal hernias included, representing 2.7% of all inguinal hernias reported. Scrotal hernia patients had higher age, higher BMI, higher ASA score, larger defects and more risk factors but less preoperative pain than non-scrotal hernia patients. Lichtenstein was the most frequently used technique. Higher postop complication rates, complication-related re-operations and general complications (increased risks of bleeding, seroma formation, prolonged ileus or obstruction, bowel injury/anastomotic insufficiency, wound healing disorder, infection) were reported after scrotal hernia repair. However, less chronic postoperative pain was seen at 1 year.

### Specialization

#### KQ3: In View of Increased Risks of Complications in Repairing Scrotal Hernias (Open or Endoscopic)—Should these be Performed by “Dedicated” Specialist Hernia Surgeons?

Recommendation: Management of scrotal hernias (especially for example S2 and S3/ Irreducible/Loss of Domain/ASA 4) can be associated with a high degree of complexity and thereby a high risk of complications. It is suggested that repairs in these patients be performed by teams that include dedicated Abdominal Wall Hernia surgeons.

Level of Evidence: Very Low.

Strength of recommendation: Weak.

Discussion: Köckerling et al. (5) demonstrated, with data from the HerniaMed Registry, that scrotal hernias have a higher postoperative complication rate, higher complication related re-operations and more general complications. Matthews et al (16) reported results of 2164 men enrolled in Veterans Affairs Cooperative study with 1983 undergoing randomly assigned surgical repairs (laparoscopic vs. open) with 2-year minimal follow-up. In both types of repairs, scrotal (and recurrent) hernias were more likely to result in complications than primary hernias. These findings support the assertion that large (and recurrent) hernias are more complex to repair and result both in higher rates of hernia recurrence and complications**.**

Leibl et al. (6) reported a series of 191 scrotal hernias including 22% with recurrence. The operating time was 65 min for reducible and 68.5 min for irreducible compared to 45 min for “non-complex” hernias. Bittner et al. (8) performed an analysis of 440 scrotal hernias in a large single-center series of 8,050 TAPP repairs. The overall recurrence rate was 0.7%, but 2.7% for scrotal hernias.

Bansal et al. (17) reported results from a retrospective study of 144 patients with scrotal hernia, including 10 with massive hernias. All patients had a urinary catheter inserted preoperatively. There were 5 unilateral orchidectomies, 6 serosal tears requiring laparoscopic repair, 4 bladder injuries identified intra-operatively and repaired laparoscopically. A total of 21 patients developed urinary retention and 42 patients developed post-operative seroma formation. Totally extra-peritoneal (TEP) repair was successful in 75.3% and transabdominal preperitoneal (TAPP) in 89.8% of patients in this case series. Whether to do TEP or TAPP depended on surgeon’s expertise. They however recommended TAPP instead of TEP for irreducible scrotal hernias and TEP for reducible hernias.

In summary, these studies indicate that scrotal hernias take longer to repair and are associated with more intra-operative and post-operative complications (bowel and bladder injuries, testicular atrophy, bleeding, seroma formation and recurrence). A high level of awareness, personal commitment and high expertise is suggested when attempting to treat scrotal hernias. In scrotal hernia factors like irreducibility, recurrence, loss of domain, S2 and S3 (Giant), ASA 4, and patient frailty increase complexity and may be better managed by teams that include dedicated Abdominal Wall Hernia surgeons. As there is no worldwide accepted definition of a Hernia Specialist HerniaSurge suggests that experienced and dedicated hernia surgeons perform scrotal hernia repairs.

### Watchful Waiting

#### KQ4: Is there a Role for Watchful Waiting in Scrotal Hernias?

Recommendation: Despite the higher rate of complications, scrotal hernias are suggested to be repaired since quality of life improvements and decreased post-operative chronic pain scores have been demonstrated.

In high resource countries, in consideration of comorbidities in this patient cohort, watchful waiting can be considered after shared decision between surgeon and patient.

In low resource countries, with limited access to acute care surgery, watchful waiting is not suggested.

Level of Evidence: Very Low.

Strength of recommendation: Weak.

Discussion: There are no papers specifically reporting on the role of watchful waiting in scrotal hernias. Scrotal hernias dramatically impair patients’ quality of life and their size is responsible for difficulty in walking, sitting or lying down, resulting in restricted mobility and limitations on activities of daily life. Patients can also suffer from voiding difficulty as the penis becomes “buried” in the scrotal fold. This causes urine to dribble over the already stretched and thin scrotal skin which in turn can lead to ulceration and secondary infections. All have negative impacts on the quality of life that can be eliminated by repair. Jacob et al. (18) reported less chronic postop pain at 1 year after scrotal hernia repair irrespective of whether the repair was performed by open or endoscopic technique.

Scrotal hernias are more common in low resource countries. They also present at a younger age and thereby cause a significant economic burden as men with symptomatic hernias can no longer support themselves and their families financially. Sanders et al. (7) reported the prevalence of inguinal hernia in Ghana to be 7.7% of the male population. The mean age for diagnosis was 34 years in Ghana compared to 62 years in the UK. Yet 67% of hernias in Ghana were scrotal compared to 6% in the UK. A total of 16% of patients in Ghana were unable to work due to their hernia symptoms. Osifo et al (3) reported on 134 patients with scrotal hernias aged 13–70 (mean 32) years in Ghana. This accounted for 51.3% of adults being treated for groin hernia. The average duration of symptoms was 14.5 years, with prolonged time to receiving care most attributed to lack of awareness in 61.2% and financial constraint in 25.4%. More importantly, two patients presented during the study period with strangulation, bowel gangrene and endotoxic shock and died after surgery.

For these reasons, unlike standard inguinal hernias where watchful waiting is acceptable for those with minimal or no symptoms, it is suggested that all scrotal hernias be electively repaired in a timely manner. Watchful waiting is an option after shared decision with the patient in high resource countries. In low resource settings where acute surgery in cases of strangulation is less available, there is an added argument for timely repair of scrotal hernias.

### Diagnostics

#### KQ5: Should Preoperative Diagnostic Imaging be Requested for Scrotal Hernias?

Recommendation: Reducible scrotal hernias do not require any pre-op imaging while irreducible scrotal hernias are suggested to undergo pre-op cross-sectional imaging or ultrasound if available.

Level of Evidence: Very Low.

Strength of recommendation: Weak.

Discussion: As per HerniaSurge guidelines, no radiological imaging is required for standard inguinal hernias including reducible scrotal hernias. However, cross-sectional imaging in the form of CT scan or MRI is beneficial in cases with irreducibility to assess the nature of the herniated content as herniated bowel loops or bladder represent increased risks for visceral injury as compared to herniated omentum (15, 17). Left sided scrotal hernias often present with colon/sigmoid herniation, frequently of the sliding type. Right sided scrotal hernias tend to contain omentum or small bowel loops. Furthermore, US in low resource countries can differentiate hydrocele versus hernia and bowel versus omentum, which might impact with the decision to operate.

### Anesthesia

#### KQ6: What is the Role of Local Versus Regional Versus General Anesthesia in Scrotal Hernia Repair?

Statement: General anesthesia must be used for endoscopic approaches for scrotal hernias. For anterior approaches, local anesthesia can be used in large reducible scrotal hernias in selected cases, especially in low-resource countries, although the risks of requiring intra-venous sedation or conversion to regional or general anesthesia should be considered.

Level of Evidence: Very Low.

Recommendation: General or regional anesthesia is recommended for irreducible scrotal hernias.

Regional anesthesia is not generally recommended due to a higher risk of urine retention and necessity of a Urinary Catheter. However, in low resource settings it is often the only option.

Level of Evidence: Very Low.

Strength of recommendation: Weak.

Discussion: Endoscopic inguinal hernia repair requires general anaesthesia as per HerniaSurge guidelines (1). Local anesthesia can be used for scrotal hernia repair *via* an anterior approach for both mesh (7) and suture repair (19). The latter demonstrated feasibility of repair of even S3 hernias under local anesthesia although there is a small risk of failure that could require intravenous sedation, regional or general anesthesia. This risk is less when using a Transversus Abdominis Plane block 30 min before local anesthetic injection. Osifo OD et al. (3) reported in a prospective study of 134 patient undergoing tissue repair for scrotal hernia under Lidocaine local anesthesia with only 10 patients requiring IV sedation. The HerniaSurge guidelines and updates suggest to use either General or Local, especially in frail patients (1). Regional (non-availability of general anesthesia in low resource regions) in some instances is the only option.

### Suture Repair

#### KQ7: Is there a Role for Suture Repair in the Management of Scrotal Hernia?

Recommendation: In general, and consistent with the inguinal hernia guidelines, the use of mesh is recommended in the repair of scrotal hernias. However, in low resource settings and in cases of contamination, a suture repair may be considered.

Level of Evidence: Very Low.

Strength of recommendation: Weak.

Discussion: HerniaSurge guidelines recommend mesh repair for L3/M3hernias. However, in low resource countries, if mesh is not available, and in cases of contamination, Shouldice suture repair is recommended using a non-absorbable monofilament suture. Non-licensed mesh is only suggested if there is no commercial mesh available (1).

### Operative Techniques (Excluding Suture Repair)

#### KQ8: What is the Recommended Technique for Repair of a Scrotal Hernia?

Recommendation: Depending on expertise, minimally invasive techniques can safely be employed. Although laparoscopic options are feasible, open repair remains the default operation for irreducible scrotal hernias. It is suggested that surgeons treating scrotal hernias are proficient in both anterior and posterior approaches.

Level of Evidence: Very Low.

Strength of recommendation: Weak.

Discussion: Endoscopic inguinal hernia repair rates have approached some 70% in one large registry yet for scrotal hernia repair (5) in the same countries the anterior approach is used in around 75% of cases, suggesting that it remains the default operation. A specialized hernia center reported TAPP repair in 193 patients with scrotal hernia with 2 recurrences after a follow-up of 30 months (6). Similarly, Bansal et al. (18) reported endoscopic repair in 144 patients with scrotal hernia with good results. TEP was used in 85 and TAPP in 59 patients although 25 of the TEP and 18 of the TAPP group required a hybrid laparoscopic-assisted approach where a 3–4 cm incision was made over the hernia sac for adhesiolysis of sac contents and removal of the sac followed by skin closure before completing the procedure laparoscopically. As few articles describe TAPP/TEP for scrotal hernia and these report a high conversion rate even with expert surgeons, HerniaSurge suggests open repair to be the standard technique. As stated in HerniaSurge guidelines, surgeons in these cases should be proficient in both anterior and posterior approaches (1).

### TEP or TAPP

#### KQ9: For Patients Considered Suitable for Endoscopic Repair Which Method, TEP or TAPP, is More Suitable?

Statement: In expert hands, it seems safe to use either the TEP (25% conversion rate) or TAPP techniques for scrotal hernia repair. TAPP is the safest minimally invasive approach for irreducible scrotal hernias. Conversion and complication rates are high and depend on the size and complexity of the hernia.

Level of Evidence: Very Low.

Discussion: Leibl et al. (6) described successful TAPP repair of 191 scrotal hernias with a recurrence rate of 1%. For irreducible scrotal hernias there are specific maneuvers which can be attempted to reduce the sac.

Ferzli et al. (20) reported TEP repair in 17 patients with a giant scrotal hernia. The authors routinely divided the inferior epigastric vessels to allow access to the deep internal ring without risk of injury to these vessels. It also permitted the release of the transversalis fascial sling, which allowed division of the floor toward the external ring. This maneuver allowed safe reduction of the large indirect sac in most cases. Also, by approaching the deep internal ring along the antero-lateral aspect it avoided injury to cord structures and allowed identification of any preperitoneal cord lipoma. Bansal et al. (17) successfully repaired scrotal hernias using TEP in 75% of cases albeit with a hybrid laparoscopic assisted approach in some cases. Of TEP repairs, 25% were converted to TAPP, while TAPP was successful in 90% of cases. Whether to do TEP or TAPP depended on surgeon’s expertise. They however recommended TAPP instead of TEP for irreducible scrotal hernias and TEP for reducible hernias.

TEP may be employed safely with expertise, but one should have low threshold to convert to TAPP or open if technically not feasible. TAPP is the safest MIS approach for irreducible scrotal hernias (5). The default operation for irreducible scrotal hernia is the anterior approach (Lichtenstein).

### Prophylactic Antibiotics

#### KQ10: Is routine preoperative antibiotic prophylaxis necessary in scrotal hernia repair?

Recommendation: While there is generally no indication for antibiotic prophylaxis for primary inguinal hernia repair (whether anterior or endoscopic), scrotal hernias represent a higher risk cohort often with prolonged operative duration, increased dead space and tissue manipulation, and potential bowel involvement as well as urinary catheterization. Pre-operative IV antibiotic prophylaxis is recommended irrespective of the hospital setting (high or low resource countries).

Level of Evidence: Low.

Strength of recommendation: Upgrade to Strong.

Discussion: HerniaSurge guidelines and updates state that especially in laparo-endoscopic repairs, but also in primary inguinal hernia repairs prophylactic antibiotics are not indicated (1). Scrotal hernias have a higher complication risk and do not fit in the category of patients in which RCT’s offered the high level evidence that antibiotic prophylaxis is not indicated. HerniaSurge recommends using antibiotic prophylaxis when performing scrotal hernia repair.

### Urinary Catheterization

#### KQ11: Should Urinary Catheterization be Routinely Recommended in Scrotal Hernia Repair?

Recommendation: Bladder catheterization is not routinely recommended in scrotal hernia repair. In complex scrotal hernia, (irreducibility, recurrence, loss of domain, S2 and S3, ASA 4, frail patients), TEP/TAPP approach, and in situations with anticipated long operative time, catheterization is suggested.

Level of Evidence: Very Low.

Strength of recommendation: Upgrade to Strong.

Discussion: The operating time for scrotal hernia is longer compared to a standard inguinal hernia (5, 6) and as the urinary bladder fills during surgery, this will place it at increased risks of injury also during anterior approach. For endoscopic approach, urinary catheterization does not negate the risks of bladder injuries (17). For the endoscopic approach, the decision on whether to catheterize or not depends on reducibility of the scrotal hernia. For irreducible scrotal hernias, it is recommended (upgraded by HerniaSurge) that patients are catheterized to reduce the risks of bladder injury. Furthermore, catheterization will allow easier and safer dissection of the space of Retzius which will also improve proper mesh placement. For patients with a reducible scrotal hernia undergoing endoscopic repair, it is suggested that patients are catheterized (upgraded by HerniaSurge), especially for S2 and S3 hernias, although this depends on the experience of the surgeon as well as patient factors such as relative advanced age with potential for incomplete preoperative bladder emptying due to prostatic hypertrophy. Furthermore, for patients undergoing TEP repair for scrotal hernia (reducible or irreducible) it is suggested (upgraded by HerniaSurge) that patients are catheterized to enable safer insertion of trocars, reduce the risks of bladder injury during dissection of the space of Retzius, to improve mesh placement and potentially reduce the risks of post-op urinary retention, especially if the operation is prolonged. Bansal et al. (17) reported 5 patients (of 144) with bladder injuries that were repaired laparoscopically, despite the fact that patients were routinely catheterized. Furthermore, 15% of patients developed urinary difficulties after the catheter removal.

### Reducible Versus Irreducible Scrotal Hernia

#### KQ12: Does the Management Differ for Reducible Versus Irreducible Scrotal Hernia?

Statement: It is important to note that many irreducible scrotal hernias can be reduced after induction of general anesthesia and muscle relaxation as well as placing the patient in Trendelenburg position. Consequently, the decision as to whether the surgeon decides on an anterior or laparoscopic approach may not be made until attempted reduction of herniated content has been initiated. Even for experienced laparoscopic surgeon’s irreducibility may sway them toward an open anterior approach if they are only experienced in the TEP approach.

Level of Evidence: Very Low.

Discussion: Many irreducible scrotal hernias reduce after induction of anesthesia, whether this be general or regional (see KQ6). They can then be treated as reduced hernias. Irreducibility (during start of procedure) increases the operation time (5, 6) and is usually associated with increased complexity including sliding hernias, involvement of abdominal viscera and/or a large amount of omentum. The latter may need to be excised to enable reduction. Hence general anesthesia is recommended, certainly in cases where TAPP/TEP is considered (17). Surgeons with limited TAPP/TEP skills may select to perform an anterior repair if attempted sac reduction under general anesthesia is unsuccessful. The patient needs to be appropriately consented for this eventuality pre-operatively. Surgeons in low resource settings without possibility of general anaesthesia are suggested to perform an operation for irreducible scrotal hernia under regional anesthesia if they are not comfortable and/or experienced in the use of local anesthesia in this cohort.

### Sac Management

#### KQ13: What is the Ideal Management of the Sac?

Statement: While it is preferrable to attempt to reduce the sac *en bloc*, there are occasions when this is not possible or even necessary. The decision of whether to abandon the sac depends on difficulties encountered during attempted sac reduction and surgeon’s preference. Transecting the neck and leaving the distal sac *in situ* probably increases the risk of seroma formation.

Level of Evidence: Very Low.

Discussion: Morrell et al. (21) described the Primary Abandon-of-the-Sac (PAS) technique during TAPP repair where the neck of the defect is incised circumferentially with the sac being left *in situ*. The inferior peritoneal flap can then be dissected as intended and the peritoneal flaps can be closed with continuous suture. Ferzli GS et al (20) routinely ligated the inferior epigastric vessels to allow safe enlargement of the internal ring in the antero-medial direction which permitted complete sac reduction in most cases. Daes (22) described an eTEP technique in 6 patients with scrotal hernias ligating the proximal end and edges of distal sac pulled up and fixated lateral to the posterior inguinal canal 5–7 cm superior to the ilio-pubic tract to avoid seroma formation. This was assisted by lowering insufflation pressure, pulling the testis down and external pressure to ipsilateral scrotum with care taken to avoid cord structures. Savoie et al. (23) reported a prospective study of 25 scrotal hernias undergoing the Bassini repair where the sac was left *in situ* with the cord structure and testis preserved. There were no recurrences but three seromas with two requiring aspirations. There is very little evidence concerning the safety and value of transecting the sac but many experienced surgeons that have performed hundreds of sac transections suggest to do it in selected cases, especially with very large scrotal sacs.

### Drains Use

#### KQ14: Does the Use of Drains Decrease the Incidence of Seroma Formation in the Repair of Scrotal Hernia, Open or Endoscopic?

Statement: For standard inguinal hernia repair, either open or laparoscopic, HerniaSurge does not recommend the use of drains. However, for scrotal hernias the use of drains may be justified depending on surgeon’s preference and patient factors such as large size or S2/3 (outside the focus of this guideline) hernias.

Level of Evidence: Very Low.

Discussion: Theoretically, any decrease in the dead space in the residual sac or space should decrease the risks of seroma and hematoma formation. However, the risks of the latter will be reduced by meticulous dissection and attention to hemostasis in both open and endoscopic repair (5). In endoscopic repair, closed suction drains can be brought out through one of the port sites. Irrespective of whether drains are used or not, the risks of seroma formation can be up to 29% (17). Agresta et al. (24) reported a series of 10 bilateral scrotal hernias treated by TAPP repair where scrotal drains were used in all patients and removed after 3 days. No seroma formation was reported in any patient. Most seromas are asymptomatic and will resorb within 3 months (25). A small percentage may require aspiration a few times before they resolve (5, 17). Should a seroma remain large and symptomatic, surgical intervention is warranted at a later stage.

### Orchidectomy

#### KQ15: Should Orchidectomy be Considered in the Operative Management of Scrotal Hernias?

Statement: Orchidectomy may be considered for complex or longstanding scrotal hernia but is not routinely recommended. The possible necessity should be considered in the informed consent process.

Level of Evidence: Very Low.

Discussion: Leibl et al. (6) reported 1 case of testicular atrophy from a series of 193 patients undergoing TAPP scrotal hernia repair, Bansal et al. (17) reported 5 unilateral orchidectomies during endoscopic repair of 144 scrotal hernias and Osifo et al. (3) reported a series of 134 patients with scrotal hernia undergoing anterior repair with no orchidectomy performed. Orchidectomy is generally not necessary in scrotal hernia repair although it is occasionally performed due to acute ischemia post-operatively which can be confirmed by doppler ultrasound. Orchidectomy has medico-legal implications in terms of adequate informed consent of its possibility.

### Cremaster Resection

#### KQ16: What is the Ideal Management of the Cremaster and Nerves in Scrotal Hernia Repair?

Statement: In open anterior repair, nerve identifying surgery and pragmatic resection are recommended. In longstanding scrotal hernias with enlargement of the cord, hypertrophic cremaster and an enlarged internal ring a cremaster resection and pragmatic neurectomy can be necessary to reconstruct the internal ring.

Level of Evidence: Very Low.

Discussion: In open anterior repair, nerve identifying surgery and pragmatic resection are recommended (1). The anatomy can be distorted in scrotal hernia, including hypertrophic cremaster which includes and protects the ilioinguinal nerve and genital branch of the genito-femoral nerve. This can necessitate resection so more frequent nerve resection is expected.

### Scrotal Skin Management

#### KQ17: What is the Role of Scrotoplasty in the Management of Scrotal Hernias?

Statement: The scrotal skin has an unusual ability to stretch and shrink and it is rarely necessary to excise the excess skin during open or endoscopic scrotal hernia repair unless the skin is compromised.

Level of Evidence: Very Low.

Discussion: Of the 134 patients undergoing Bassini repair for scrotal hernia, no patient underwent scrotoplasty although 18 patients developed scrotal hematoma, most of which resolved spontaneously within 3 months (3). Similarly, Bansal et al. (17) did not report any scrotoplasty in their series of 144 scrotal hernias undergoing endoscopic repair. Unless the scrotal skin is compromised there is no need for scrotoplasty during open or endoscopic repair. Delayed scrotoplasty can be considered in cases with persistent redundancy and/or troublesome scrotal hematoma.

### Mesh Fixation

#### KQ18: Should Mesh Fixation be Recommended for all Endoscopic Scrotal Hernia Repairs?

Statement: As per HerniaSurge guidelines, in endoscopic repair direct inguinal and hence scrotal hernias with large defects should have adequate overlap across the midline and the mesh should in M3 defects be fixed with traumatic fixation in the midline and/or pubic ramus to minimize the risks of a direct recurrence. A larger and “heavier” weight mesh should be considered for very large defects.

Level of Evidence: Very Low.

Discussion: Reduction of a large direct sac and decreasing the dead space, either with tack inversion to Cooper’s ligament or the rectus abdominis or the use of an Endoloop to imbricate the transversalis fascia, will minimize the risks of the medial aspect of the mesh eventrating into the defect. For large indirect defects, the sac can be inverted and tacked supero-laterally well above the inguinal nerves to reduce the risks of seroma formation and recurrence (22). It is not recommended to narrow the defect with suture because of the risks of nerve entrapment. It is recommended (upgraded by HerniaSurge) to use a larger mesh and consider a heavier weight prosthetic to adequately cover the defect.

### Management of Hydrocele and Scrotal Hernia

#### KQ19: What is the Management of a Coexistent Hydrocele at Scrotal Hernia Repair?

Recommendation: In view of increased rate of complications including infection, concomitant treatment of hydrocele with scrotal hernia repair is not suggested.

Level of Evidence: Very Low.

Strength of recommendation: Weak.

Discussion: In a study from Haiti (26), the rates of infectious complications increased with concurrent hydrocele or haematocele repair. In general, the treatment of hydroceles or haematoceles may be separated from the hernia repair.

## Discussion

The highest available evidence for scrotal hernia repair is level 2 (2 articles) with most current research articles being case series with relatively small number of patients included. No prospective randomized controlled studies have been identified. Given the relatively small numbers of scrotal hernias seen in high resource countries compared to lower resource countries, it is difficult to draw any firm conclusions for their treatment. High level research is challenging and an RCT is probably not feasible.

Anterior suture repairs are generally not recommended in high resource countries where the use of mesh reinforcement reducing the risk of a recurrence has long been advocated. Despite this, in low resource settings either because of a lack of training or resources (unavailability or unaffordable mesh), mesh techniques are rarely used.

The true results of suture repairs in low resource settings are not known. A well conducted study with long-term follow-up comparing mesh to suture repair in scrotal hernia should be encouraged. There is a distinct difference in the average patient and hernia characteristics in many low resource countries compared to high resource countries (for example average age, bodyweight). Given the high prevalence of scrotal hernias in such settings (2) it would not take long to obtain a large patient enrollment to conduct either a well-designed RCT or a prospective registry study. The null hypothesis would be that a standardized suture repair is as good as a mesh repair concerning recurrence within 3 years, when compared to mesh repairs. Secondary outcomes would evaluate infectious complications and reoperations for short term recurrence or infection. Quality of life is of great importance and if this can be improved after scrotal hernia surgery in a low resource setting, the added operative complexity and risk is justified. The main question to be answered: “Can we help this group of young men to achieve a better quality of life by repairing their hernias without causing them new harm?”

Given a significant number of humanitarian hernia missions around the world (7, 19) where donation and hence availability of mesh is common, RCTs comparing mesh vs. non-mesh repairs in scrotal hernia could be considered. Involvement of local surgeons will be paramount, not only from the perspective of imparting standardized techniques and encouraging academic pursuits, but also in allowing for long-term follow-up of patients. An important factor is adequate training for surgeons (and medical officers) that perform inguinal hernia repairs.

## Training and Goals

The average general surgeon performs around 28 inguinal hernia repairs annually (1). Since scrotal hernias represent only 2%–6% of all inguinal hernias (2, 17), there is a challenge for surgeons in obtaining sufficient experience to deal with even S1 hernias let alone or S2/3 with or without reducibility. Consequently, dedicated surgeons with special interests in hernia surgery are likely to provide a more comprehensive tailored treatment. Therefore, general surgeons should feel comfortable in liaising with or referring patients to more experienced colleagues in providing the best treatment for (complex) scrotal hernias depending on competence and resource capabilities. It is noteworthy that, in 2021, the European Union of Medical Specialists (UEMS: Union Européenne des Médecins Spécialistes), under the leadership of Professor Ferdinand Köckerling, took important step in recognizing abdominal wall surgery (AWS) as a sub-specialty of general surgery accrediting qualified surgeons as Fellow of the European Board of Surgery, Abdominal Wall Section (FEBS AWS)^1^.

While the HerniaMed Registry (5) demonstrated that scrotal hernias have a higher postoperative complication rate, higher complication related re-operations and more general complications, it is a voluntary database representing only a select group of hernia centers and hospitals. In contrast, the Danish Hernia Database (27) is a nationwide compulsory registry including all patients undergoing inguinal hernia repair since 1997. Unfortunately, it does not differentiate scrotal hernia as part of the registration form. As there is no universally accepted classification for scrotal hernias, it is hoped that this proposed simple scrotal hernia classification will be adopted worldwide to better compare and tailor different treatment options for different scrotal hernia types (S1-3).

In high resource settings, an open anterior repair is the default operation. The Lichtenstein operation is still considered the gold standard for anterior open repair (1). The endoscopic hernia repair methods have been shown to be safe and effective with acceptable low complication rates in specialized centers (5, 15, 17, 20). There is a high conversion rate when starting with an endo-laparoscopic technique, especially TEP. Low resource countries may not be able to afford the mesh and/or consider their operative settings to be sufficient for sterile standards to prevent mesh infection and its sequelae. Therefore, suture repair still remains a standard option in these settings. Teaching and training to master the Shouldice technique remains an important cornerstone for surgical management of inguinal hernias in low resource settings.

## Conclusion

Scrotal hernias account for around 67% of all inguinal hernias treated in low resource countries, compared to 2% in high resources countries. Patients in low resource countries are affected at a much younger age, on average in the fourth decade of life. Yet, this is the age group where up to 17% of those afflicted cannot work because of their hernia with a consequent personal and societal financial burden. The negative impact on quality of life seen in all age groups means that scrotal hernias should in general be repaired in a timely manner. This chapter provides guidance to surgeons from diagnostic imaging, operative repair techniques to prevention of complications. This guideline is by intention general in its design and content. It can be used by surgeons of all abilities and from different resource settings with the aim to maximize surgical patient outcomes in this challenging cohort.

## HerniaSurge Collaboration

F. Agresta, F. Berrevoet, I. Burgmans, D. C. Chen (AHS), A. de Beaux, B. East, N. Henriksen, F. Köckerling, M. Lopez-Cano, R. Lorenz, M. Miserez, A. Montgomery, S. Morales-Conde, C. Oppong, M. Pawlak, M. Podda, D. Sanders, A. Sartori, M.P. Simons (former EHS secretary for quality), C. Stabilini (EHS secretary for Science), H. M. Tran (Australasian Hernia Society), N. van Veenendaal, M. Verdauguer, R. Wiessner.

## Data Availability

The original contributions presented in the study are included in the article/supplementary material, further inquiries can be directed to the corresponding author.

## Footnotes

1
https://www.europeanherniasociety.eu/uems (Accessed Feb 13, 2023).
